# Visuospatial outcomes of a prospective national cohort of young adults with very low birthweight

**DOI:** 10.1038/s41390-025-03890-9

**Published:** 2025-02-07

**Authors:** Sarah L. Harris, Lianne J. Woodward, L. John Horwood, Tracy R. Melzer, Samudragupta Bora, Maddie Pascoe, Brian A. Darlow

**Affiliations:** 1 Department of Paediatrics, University of Otago, Christchurch, New Zealand; 2 School of Health Sciences & Canterbury Child Development Research Group, University of Canterbury, Christchurch, New Zealand; 3 Department of Psychological Medicine, University of Otago, Christchurch, New Zealand; 4 New Zealand Brian Research Institute, Christchurch, New Zealand; 5 Department of Medicine, University of Otago, Christchurch, New Zealand; 6 School of Psychology, Speech and Hearing, University of Canterbury, Christchurch, New Zealand; 7 Department of Pediatrics, University Hospitals Rainbow Babies & Children’s Hospital, Case Western Reserve University School of Medicine, Cleveland, OH USA

## Abstract

**Background:**

Visuospatial processing is reportedly impaired in children born very preterm (VP) compared with full term (FT) controls but there are few data for VP adults.

**Methods:**

At 26-30 years, 225 very low birthweight (VLBW) adults (70% national cohort survivors) and 100 FT controls were assessed on motor-dependent visuospatial skills using the Block Design subtest of the Wechsler Adult Intelligence Scale, and nonmotor-dependent skills by the Benton Judgment of Line Orientation and Brixton Spatial Anticipation tests. A composite score was created by summing standardized scores for the three tests. MRI measures of cortical volume, thickness and surface area were obtained for 150 VLBW participants.

**Results:**

VLBW born adults performed less well than controls across all visuospatial measures and their composite score (*P* < 0.001), with moderate to large effect sizes (Cohen’s *ds* = 0.41–0.82). Between group differences were not explained by current vision impairment, cerebral palsy, sex, ethnicity or socio-demographic factors. The unadjusted visuospatial composite score was significantly correlated with reduced cortical surface area and cortical volume, but few correlations remained significant after adjustment for age, sex and intracranial volume.

**Conclusion:**

The visuospatial functioning of adults born VLBW is significantly poorer than their FT peers with only modest associations with cortical brain structure.

**Impact:**

- Previous reports have shown very preterm children have impaired visuospatial processing compared with term-born peers but only limited data address whether these impairments persist into adulthood.
- Visuospatial functioning, assessed by both motor and non-motor dependent tests, of adults born very low birthweight is significantly poorer than that of term-born peers.
- Poorer visuospatial functioning in this very low birthweight cohort is not explained by vision impairment and had only modest associations with cranial MRI brain structure.
- Persisting visuospatial impairment in very preterm adults may significantly impact quality of life. Early recognition of these difficulties could facilitate support strategies to improve outcomes.

## Introduction

Visuospatial functioning is a key component of cognition that involves the ability to perceive objects in the environment, and accurately assess distances between objects and in relation to self.^1^ It allows an individual to perceive the speed of moving objects and plays a role in everyday activities, such as recognizing faces, dressing, reading, writing, playing sport, riding a bike, driving a car and safely navigating the environment.^2^ Children born very preterm (VPT, <32 weeks’ gestation) and/or very low birth weight (VLBW, <1500 grams) are at increased risk of a range of visual impairments, the most significant of which is retinopathy of prematurity and its sequelae.^3–10^

VPT children are also at increased risk of visuospatial processing impairments.^11–15^ A meta-analysis of 16 studies found that VPT/VLBW children perform less well on measures of visuospatial processing compared to their term-born peers or population norms, with combined effect sizes (Cohen’s d) ranging from 0.60 to 0.92 (moderate to large).^13^ However, results have not been consistent across measures, with methodological differences in study sample selection, age at assessment and tools used, further limiting interpretation of existing study findings. Also, little consideration was given to the effects of visual acuity and other potentially confounding neurodevelopmental factors. Limited data also exist on the extent to which these impairments persist into adulthood.^16–18^

Visuospatial functioning is dependent not only on visual perception but also motor skills and involves complex neural networks spanning multiple brain regions.^19–22^ In MRI studies of other adult populations subject to visuospatial impairment, including those with Parkinson’s disease and dementia, regional cortical thinning, particularly in the parietal and temporal regions has been linked with visuospatial abilities.^23,24^ There is, however, a paucity of adult MRI data in populations who have had fetal brain maturation interrupted by premature birth.^17^

The New Zealand Very Low Birthweight Study is a population-based longitudinal cohort born VLBW in 1986 who had comprehensive visual and neurocognitive assessment and concurrent brain MRI at age 26-30 years.^7,25,26^ The aims of this paper were to:

1. Examine the motor and nonmotor-dependent visuospatial functioning of this cohort relative to a term-born, appropriate birth weight control group and
2. Identify, within the VLBW group, structural brain MRI correlates (cortical thickness, surface area and volume) of adult visuospatial function.

We hypothesised that, compared to adults born full term (FT), VLBW adults would have poorer visuospatial functioning and that this would be associated with brain metrics in brain regions involved in visuospatial processing.

## Methods

### Sample

The VLBW cohort included all 413 infants born VLBW admitted to a neonatal unit in New Zealand in 1986, with 78% (323/413) surviving to hospital discharge. Of these infants, 250 completed a previously reported visual and neurodevelopmental follow-up assessment between ages 26 and 30 years.^7,25,26^ The mean (SD) age at assessment was 28.4 (1.1) years for VLBW adults and 28.2 (0.9) years for FT peers. Sample retention was 77% (250/323 survivors), with data from 225 young adults available for this analysis (Supplemental Fig. S1, online).

For comparison purposes, a group of 100 age-matched term-born young adults was recruited either through peer nomination by a cohort member (*n* = 76) or random sampling from electoral rolls (*n* = 24), aiming to ensure balance with respect to infant sex, ethnicity, and region of birth: 39 were recruited at age 22–23 years and 61 at 26–30 years (Supplemental Fig. S1, online).

Study procedures were approved by the Upper South B Regional Ethics Committee and written informed consent was obtained from all participants.

### Measures

#### Visual and visuospatial assessment

Visual assessment included distance visual acuity using standardised ETDRS chart (with glasses if worn), glasses prescription-measured in focimeter, contrast sensitivity testing with Pelli–Robson charts (which reflects functional visual ability), autorefraction and retinal photography. Moderate or worse vision impairment was defined as: any visual acuity >0.3 LogMAR (poorer than Snellen 6/12), myopia >2 D, hypermetropia >2 D or astigmatism >2 D.^7^

Visuospatial outcomes were assessed using three standardized tests encompassing both motor and nonmotor skills as described below. Across all measures, test trials increased in complexity.

Motor-dependent visuospatial skills were evaluated using the *Block Design subtest of the Wechsler Abbreviated Scale of Intelligence®* – Second edition (WASI-II),^27^ primarily assessing *visuospatial judgment and visuomotor integration*. This timed test required participants to manipulate a series of three-dimensional red, white, or mixed-coloured blocks to reproduce a model demonstrated by the assessor or illustrated in the test stimulus book.

Nonmotor-dependent visuospatial skills were evaluated using the *Benton Judgment of Line Orientation test* (BJLO),^28^ primarily assessing visuospatial judgment and localization and the *Brixton Spatial Anticipation test* (BSA),^29^ primarily assessing visuospatial judgment and sequencing. The BJLO non-timed test required participants to visually judge the angular orientation of a pair of lines and to then match this to the angular orientation of a set of 11 lines arranged in a semicircle with 18 degrees separating them from each other on the same page of the stimulus book. The BSA is a non-timed visuospatial sequencing task with rule changes. Participants are required to detect nine visuospatial rules and the location of the target (blue circle) across a sequence of stimulus pages containing an array of 10 circles (two rows of five circles) with one blue-coloured circle per page and the position of the coloured circle shifting between pages.

A *visuospatial abilities composite score* was created by summing standardized scores across the three assessments. Mild impairment was defined as visuospatial scale score > 1 and ≤ 2 SD below the control group mean and severe impairment as visuospatial scale score > 2 SD below control mean.

#### Brain MRI measures

A subsample of 150 VLBW participants underwent an MRI scan as part of their 2-day assessment. This group consisted of VLBW adults born <28 weeks’ gestation (*n* = 53: excluding three with contraindications and one whose scan was affected by motion artefacts) and a further 97 adults randomly selected from the VLBW cohort. Images were acquired using a 3-Tesla General Electric HDxt scanner (GE Healthcare, Waukesha, WI) with an eight-channel head coil. Details of the imaging are given in our previous report,^30^ and Supplementary material (online). Thickness estimates from right and left hemispheres were averaged to create a single average thickness per lobe; total surface area and volume per lobe were also calculated.

#### Other measures

A range of perinatal clinical characteristics for VLBW adults were extracted from medical records, and measures of sociodemographic characteristics (e.g. maternal education, childhood family socioeconomic status) were assessed retrospectively in both groups via participant interview.

#### Statistical analysis

At the univariate level, between-group comparisons were tested using the independent samples t-test for comparison of means and the chi square or Fisher’s exact test for comparison of percentages. Mean between-group differences in visuospatial outcomes were adjusted for covariates using multiple linear regression: adjusting first for current vision impairment, cerebral palsy and socio-demographic factors, then additionally for verbal IQ (but not total IQ given that the Block Design is a subtest of Performance IQ). Effect sizes (ESs) were summarised by Cohen’s d^31^ for continuous measures and rate ratios for impairment measures with associated 95% confidence intervals. Within VLBW group analyses of factors associated with visuospatial function, including brain MRI measures, were conducted using multiple linear regression, with effect sizes summarised by the Pearson correlation (r) at the univariate level and the standardised regression coefficient (β) at the multivariable level. The sample had 80% power at α2 = 0.05 to detect effect sizes (Cohen’s d) > 0.33 SD between groups (VLBW vs Controls) and greater than 0.46 SD for subgroups (e.g., extremely low birth weight, ELBW vs FT). Within the VLBW group the study had 80% power to detect a correlation *r* > 0.19, and in the subgroup receiving MRI *r* > 0.23. A *p* < 0.05 was used as the threshold for statistical significance.

## Results

### Characteristics of cohort

Perinatal clinical characteristics, sociodemographic background and concurrent neurodevelopmental characteristics of adults born VLBW and FT are shown in Table 1. VLBW adults had lower birthweight and maternal education level and lower total IQ scores. A comparison of these characteristics in the surviving VLBW cohort who had visuospatial functioning assessed and those who did not is shown in Supplementary Table 1 (online). Those not assessed were more likely to have moderate or severe disability at 7–8 years of age, defined as cerebral palsy in non-ambulant children or in ambulant children causing considerable limitation of movement, or bilateral sensorineural deafness requiring hearing aids, or bilateral blindness, or an IQ score of >2 SD below the test mean (<70).

**Table 1:** Clinical, sociodemographic background and concurrent neurodevelopmental characteristics of VLBW adults and full term controls

Measure	VLBW (*N* = 225)	Controls (*N* = 100)	*p*
**Clinical Characteristics**			
Birth weight (g), *mean (SD)*	1136 (235)	3377 (584)	<0.001
<1000 g, *%*	27.1		
Gestation (wk), *mean (SD)*	29.3 (2.5)		
Expremely preterm ( < 28 wk), *%*	24.0		
Small for gestation^a^, *%*	32.0	–	
Male sex, *%*	44.4	37.0	0.21
Retinopathy of prematurity, *%*	18.7	–	
Stage 1, *%*	8.9	–	
Stage 2 or more, %	9.8	–	
Antenatal corticosteroid use, *%*	56.0	–	
Bronchopulmonary dysplasia^b^, *%*	19.6	–	
Confirmed sepsis, *%*	22.7	–	
Intraventricular haemorrhage III/IV,%	2.2	–	
**Sociodemographic Background**			
Maternal age at childbirth (y), *mean (SD)*	25.9 (5.0)	27.2 (4.5)	0.036
Mother not a high school graduate, *%*	30.7	13.0	0.001
Māori/Pacific Island ethnicity, *%*	31.1	21.0	0.06
Family socioeconomic status			
Professional/managerial, *%*	33.8	34.0	
Technical/skilled, *%*	42.7	51.0	
Semiskilled/unskilled/unemployed, *%*	23.5	15.0	0.18
**Concurrent Neurodevelopmental Characteristics**			
WASI II total IQ score, *mean (SD)*	100.3 (14.4)	111.4 (11.3)	<0.001
Verbal IQ score, *mean (SD)*	100.5 (13.8)	108.3 (11.1)	<0.001
Cerebral Palsy^c^, *%*	3.6	0.0	0.11
Moderate or worse vision impairment^d^, *%*	21.8	14.0	0.10
Age at Assessment (y), *mean (SD)*	28.4 (1.1)	28.2 (0.9)	0.19

*SD* Standard Deviation, *WASI II* Wechsler Abbreviated Scale of Intelligence 2nd edition, *LogMAR* Logarithm of the minimum angle of resolution, *D* Diopter

^a^Birth weight <10^th^ centile for gestation

^b^Oxygen requirement at 36 weeks post-menstrual age

^c^No VLBW participants tested on visuospatial abilities were diagnosed with cerebral vision impairment

^d^Any of visual acuity >0.3 LogMAR, myopia >2 D, hypermetropia >2 D or astigmatism >2 D

### Vision and visuospatial outcomes

Of the adults born VLBW 21.8% had moderate or worse vision impairment compared to 14% of adults born FT (*p* = 0.10). There was no difference in contrast sensitivity between VLBW and FT participants.^7^

VLBW young adults obtained significantly lower scores than their term born peers on all three measures of visuospatial functioning spanning both motor dependent and independent tasks (Table 2). Overall, the VLBW group scored 0.72 SD lower than FT peers on the combined visuospatial composite after covariate adjustment. Effect sizes (ESs) were in the moderate to large range (Cohen’s *d*s = 0.41–0.82), and were largely unaffected by adjustment for current vision impairment, cerebral palsy and socio-demographic factors (adjusted *d*s = 0.41–0.76). ESs reduced somewhat on additional adjustment for verbal IQ, but remained in the small to moderate ES range (adjusted *d*s = 0.24–0.55).

**Table 2:** Mean (SD) standardised^a^ visuospatial processing scores of VLBW adults and full term controls

Measure	VLBW	Controls	*P*	Cohen’s *d (95%CI)*		
				Unadjusted	Adjusted 1^b^	Adjusted 2^c^
WASI II block design score (motor)	96.2 (14.4) (*N* = 225)	108.6 (12.7) (*N* = 100)	<0.001	0.82 (0.58, 1.07)	0.76 (0.52, 1.00)	0.55 (0.31, 79)
Benton visuospatial processing score (non-motor)	97.3 (15.9) (*N* = 219)	106.0 (10.5) (*N* = 100)	<0.001	0.58 (0.34, 82)	0.57 (0.33, 81)	0.37 (0.13, 61)
Brixton visuospatial sequencing score (non-motor)	98.1 (15.7) (*N* = 213)	104.2 (12.6) (*N* = 100)	<0.001	0.41 (0.17, 65)	0.41 (0.17, 65)	0.24 (0.00, 48)
Composite visuospatial processing score	96.5 (15.2) (*N* = 211)	107.4 (11.5) (*N* = 100)	<0.001	0.73 (0.49, 98)	0.72 (0.48, 97)	0.50 (0.26, 74)

*WASI II* Wechsler Abbreviated Scale of Intelligence 2nd edition.

^a^ All outcomes standardised to mean=100, SD = 15 in the combined sample.

^b^ Adjusted for moderate or worse visual impairment, cerebral palsy, sex, ethnicity, maternal education, family socio-economic status.

^c^ Adjusted for WASI II Verbal IQ in addition to covariates listed in footnote 2.

Examination of rates of visuospatial impairment (Table 3) based on participant’s overall composite scores showed that almost half (43%) of the VLBW group compared to 16% of the FT group were performing in the impaired range, with 20% versus 5% of FT adults showing severe impairment. VLBW adults typically had a 2 to 3-fold higher risk of impairment across various visuospatial processing domains and an 18-fold higher risk for severe motor-dependent visuospatial skills despite a relatively low rate of cerebral palsy. Exclusion of VLBW with CP had negligible impact on ESs or rates of impairment (data not shown).

**Table 3:** Rates (%) of impairment on visuospatial measures of VLBW adults and full term controls

Measure	VLBW	Controls	*p*	*RR (95%CI)* of impairment
WASI II block design (motor)	(*N* = 225)	(*N* = 100)		
No impairment	54.7	85.0		–
Mild impairment	27.6	14.0		1.96 (1.16–3.34)
Severe impairment	17.8	1.0	<.001	17.78 (2.48–127.5)
Benton visuospatial processing score (non-motor)	(*N* = 219)	(*N* = 100)		
No impairment	63.5	86.0		–
Mild impairment	17.8	8.0		2.23 (1.08–4.58)
Severe impairment	18.7	6.0	<.001	3.12 (1.37–7.11)
Brixton visuospatial sequencing score (non-motor)	(*N* = 213)	(*N* = 100)		
No impairment	77.9	92.0		–
Mild impairment	8.9	3.0		2.97 (0.90–9.82)
Severe impairment	13.1	5.0	.01	2.63 (1.05–6.61)
Composite visuospatial processing	(*N* = 211)	(*N* = 100)		
No impairment	56.9	84.0		–
Mild impairment	23.2	11.0		2.11 (1.15–3.88)
Severe impairment	19.9	5.0	<.001	3.98 (1.62–9.75)

*RR* Rate ratio, *WASI II* Wechsler Abbreviated Scale of Intelligence 2^nd^ edition Mild impairment – Visuospatial scale score > 1 and ≤ 2 SD below Control mean Severe impairment – Visuospatial scale score > 2 SD below Control mean.

Further analysis examined mean visuospatial ES differences by variations in birth weight, gestation and ROP status in the VLBW group (Supplemental Table S2, online). Compared to FT peers those adults born ELBW ( < 1000 g) exhibited substantially greater impairment in visuospatial functioning (adjusted *d*s = 0.70–1.13) than adults born VLBW but not ELBW (adjusted *d*s = 0.29–0.62). A similar, but less marked pattern was observed when comparing those born extremely preterm (EPT, <28 weeks) with those born ≥ 28 weeks. ES differences by ROP stage/status, though in the expected direction, were generally modest. Additional adjustment for verbal IQ preserved the same general pattern of results but typically reduced ESs by about one third.

Supplemental Table S3 (online) shows univariate and multivariate predictors of the composite visuospatial functioning score within the VLBW group. Statistically significant predictors were birthweight, sex, socioeconomic status and cerebral palsy severity. Receipt of antenatal steroids was also a weak predictor. Jointly these factors explained 17% of the variance in composite visuospatial functioning. Of note neither a history of ROP nor concurrent visual impairment were significant predictors.

### MRI correlates of visuospatial functioning

A comparison of sociodemographic and perinatal characteristics in the surviving VLBW cohort who were assessed with MRI and those who were not is shown in Supplemental Table S4 (online). Those assessed by MRI had lower gestation and birthweight and were less likely to have neurosensory impairment. A total of 150 VLBW underwent MRI of whom 143 had data on visuospatial functioning.

Table 4 shows the associations between cortical brain metrics (average thickness, surface area and volume) and participant’s concurrent composite visuospatial scores. Separate estimates are provided by brain region (frontal, parietal, temporal, occipital) and hemisphere (left, right and total brain). At the unadjusted level, visuospatial performance was strongly correlated with all cortical volume measures (*r* = 0.27–0.39) and the majority of surface area measures (*r* = 0.17–0.32). In contrast, associations with cortical thickness metrics were much weaker (*r* = 0.04–0.21). For the most part correlations were similar across brain regions and hemispheres. Examination by the separate components of the combined visuospatial score showed: the BJLO and Block design had essentially the same pattern/size of correlations as observed in Table 4, while the BSA was generally uncorrelated with all MRI measures (data not shown).

**Table 4:** Associations (correlations) between brain metrics (average cortical thickness, surface area and volume by cortical region and hemisphere) and visuospatial composite score amongst VLBW participants with MRI (*N* = 143)

		Average Cortical Thickness			Cortical Surface Area			Cortical Volume		
Adjustment	Cortical Region	Left Hemi-sphere	Right Hemi-sphere	Total	Left Hemi-sphere	Right Hemi-sphere	Total	Left Hemi-sphere	Right Hemi-sphere	Total
Unadjusted		(r)	(r)	(r)	(r)	(r)	(r)	(r)	(r)	(r)
	Frontal	0.06	0.04	0.05	0.29***	0.29***	0.29***	0.28***	0.30***	0.30***
	Temporal	0.15	0.14	0.15	0.32***	0.25**	0.30***	0.39***	0.31***	0.36***
	Parietal	0.18*	0.16	0.17*	0.25**	0.28***	0.27***	0.32***	0.28***	0.33***
	Occipital	0.21*	0.18*	0.20*	0.17*	0.19*	0.19*	0.27***	0.32***	0.32***
Adjusted		(β)	(β)	(β)	(β)	(β)	(β)	(β)	(β)	(β)
	Frontal	0.01	0.02	0.02	0.10	0.11	0.11	0.07	0.15	0.12
	Temporal	0.07	0.06	0.07	0.19	−0.02	0.10	0.34*	0.11	0.25
	Parietal	0.14	0.12	0.13	0.00	0.06	0.03	0.17	0.15	0.18
	Occipital	0.19*	0.12	0.16	−0.09	−0.01	−0.06	0.08	0.11	0.11

^*^p < .05 **p < .01 ***p < .001

Unadjusted – Pearson correlation between brain metric and composite visuospatial score.

Adjusted – Standardised partial correlation between brain metric and composite visuospatial score from multiple regressions adjusting for age at scan, sex and (for cortical surface area and volume only) intracranial volume.

Adjustment for age at scan, sex and intracranial volume substantially reduced the strength of these associations. Adjusted associations were generally small and with two exceptions (left hemisphere temporal volume and occipital thickness) statistically non-significant. Closer examination of the two aberrant associations suggested that these were likely spurious, reflecting a ceiling effect in the assessment of the BJLO test component of the composite score, which resulted in an artificial inflation of overall associations with these two metrics. Reanalysis of the above associations excluding the BJLO test from the composite score produced estimates that were much closer to the other adjusted associations in the table.

## Discussion

We have demonstrated that compared to FT peers, visuospatial functioning is poorer and rates of visuospatial impairments are higher amongst young adults born VLBW. Visuospatial functioning performance was strongly correlated with all MRI cortical volume metrics and the majority of surface area metrics but associations were substantially reduced after adjustment for age, sex and intracranial volume. These results suggest that these structural MRI measures may not be helpful in predicting visuospatial impairment and that functional assessment of these complex neural networks may be needed in future studies.

The findings suggest that visuospatial impairments observed in children and adolescents born VLBW^13^ persist into adulthood, with moderate to large effect size differences. Those born at lower birthweights/gestations appear to be more severely affected. To date, only three studies have examined the visuospatial functioning of adults born VPT or VLBW. A prospective study of 18-22 year old VLBW adults in Norway, reported poorer visuospatial performance on both motor-dependent components of the Beery Visual Motor Integration (VMI) test, but not on the motor-independent supplemental test, compared with controls.^17^ A prospective study of young adults born VLBW in Sweden, using the VMI, reported poorer performance on all parts of the VMI test.^18^ Consistent with current findings, no correlations were found between the VMI test scores and ROP or visual acuity. A neurological disorder at age 2.5 years of age was a risk factor for lower scores on the VMI but not the visual perception subtest. At age14-20 years, an Australian population-based cohort of individuals born ELBW and/or EPT found that this group performed less well than term born controls across all five visual ability subtests of the Test of Visual Perceptual Skills (TVPS-3), including the non-motor subtest of visual-spatial relationships.^16^

The elevated risk of visuospatial impairments seen in our cohort was evident across both motor and non-motor domains, although motor-dependent skills appeared to be more compromised. This is in the context of a cerebral palsy rate of 3.6% in our sample. This may be partly attributed to the now well-documented high rates of minor neuromotor impairments in the preterm-born population, even in the absence of cerebral palsy.^32^ We did not assess minor neuromotor functioning in our cohort and so are unable to explore this association further. Nonetheless, the observed performance differences between VLBW and FT comparison groups are important due to the potential cascading impact on everyday functioning, employment, and life course opportunities.

Regional differences in cortical thickness on MRI were also found to be associated with the risk of visuospatial impairments in this population. The only other study to examine associations between brain MRI metrics and visuospatial processing in a VPT/VLBW young adult sample found poorer visual–motor integration performance was correlated with reduced cortical thickness and surface area in several brain regions.^17^ However, no adjustment was made for potential confounding factors, such as visual ability, in this study. These investigators also reported poorer VMI performance was associated with reduced fractional anisotropy in several white matter tracts, which we did not examine in our study but should be considered in future work. Subtle differences in brain structure may explain why some children born VPT or VLBW exhibit visuospatial processing deficits in the absence of overt perinatal cerebral white matter injury. Further studies with much larger samples and utilizing advanced neuroimaging techniques, such as diffusion tensor imaging or functional MRI, may be helpful in understanding the neural networks underlying visuospatial abilities.

Methodological strengths of this study include the prospective population-based cohort design with high retention from birth to young adulthood, a comprehensive battery of standardized visuospatial tests across both motor and non-motor domains, inclusion of an age-matched comparison group, and the blinding of assessors to study group status. Weaknesses include that we did not have funding to scan the whole cohort and as such those born EPT were prioritised, potentially biasing our results somewhat since these individuals were also subject to higher rates of neurological impairment. However, 97 of 143 who had MRI scans were not EPT and these participants were selected at random. We acknowledge that our construction of a composite visuospatial test score with inclusion of the BSA test, often considered to primarily assess executive function, might be considered to be a weakness. However, there is evidence that the greatest emphasis in the test is on the spatial component, highlighting the importance of visuospatial processing abilities over other core executive function skills.^33^ Additionally, the BSA test has high ecological validity for driving performance, which is heavily reliant on visuospatial abilities, a critical functional outcome for our study group. For example, performance on the BSA test was more strongly correlated with driving measures, such as reduced lane position adjustment to curve direction and decreased lane position maintenance around curves, than with commonly used executive function tests.^34^

It is unclear whether these findings will be generalisable to contemporary preterm cohorts in whom improvements in clinical care, including treatment for retinopathy and optimised nutrition, may have resulted in better neuroprotection. Further research is also needed to understand the impact of visuospatial impairments on quality of life and daily functioning in the VLBW population. More refinement of which sub-groups may be at higher risk is needed and whether, following early identification, intervention programmes can alter this risk trajectory. Concerns have also been raised that preterm birth might be associated with premature aging^35,36^ and longer follow-up is also needed to assess the impact of aging on visuospatial performance in adults born VLBW/VPT.

## Conclusion

Adults born VPT or at VLBW are at increased risk of visuospatial impairment which may significantly impact quality of life. Existing cortical brain structure measures appear to be only modestly associated with visuospatial functioning. Early recognition of these difficulties in childhood may enable proactive developmental support strategies to be offered to improve outcomes. Further research is needed to understand the impact of aging on visuospatial functioning in this vulnerable population.

## Supplementary information

Supplementary figureS1

Supplementary information

## Data Availability

The datasets generated and/or analyzed during the current study include sensitive health data and cannot be made publicly available. Aggregated data is available from the corresponding author on reasonable request.
