# Supplementary material for: Visuospatial outcomes of a prospective national cohort of young adults with very low birthweight
Source: Pediatr Res. 2025 Feb 7;98(5):1711–7. doi: 10.1038/s41390-025-03890-9 (PMC12602344; doi:10.1038/s41390-025-03890-9)
Supplement: Supplementary file 1 — Supplementary figureS1 [file 41390_2025_3890_MOESM1_ESM.pdf]

Supplemental Figure S1. New Zealand 1986 VLBW Adult Follow-up Study: Cohort flow chart

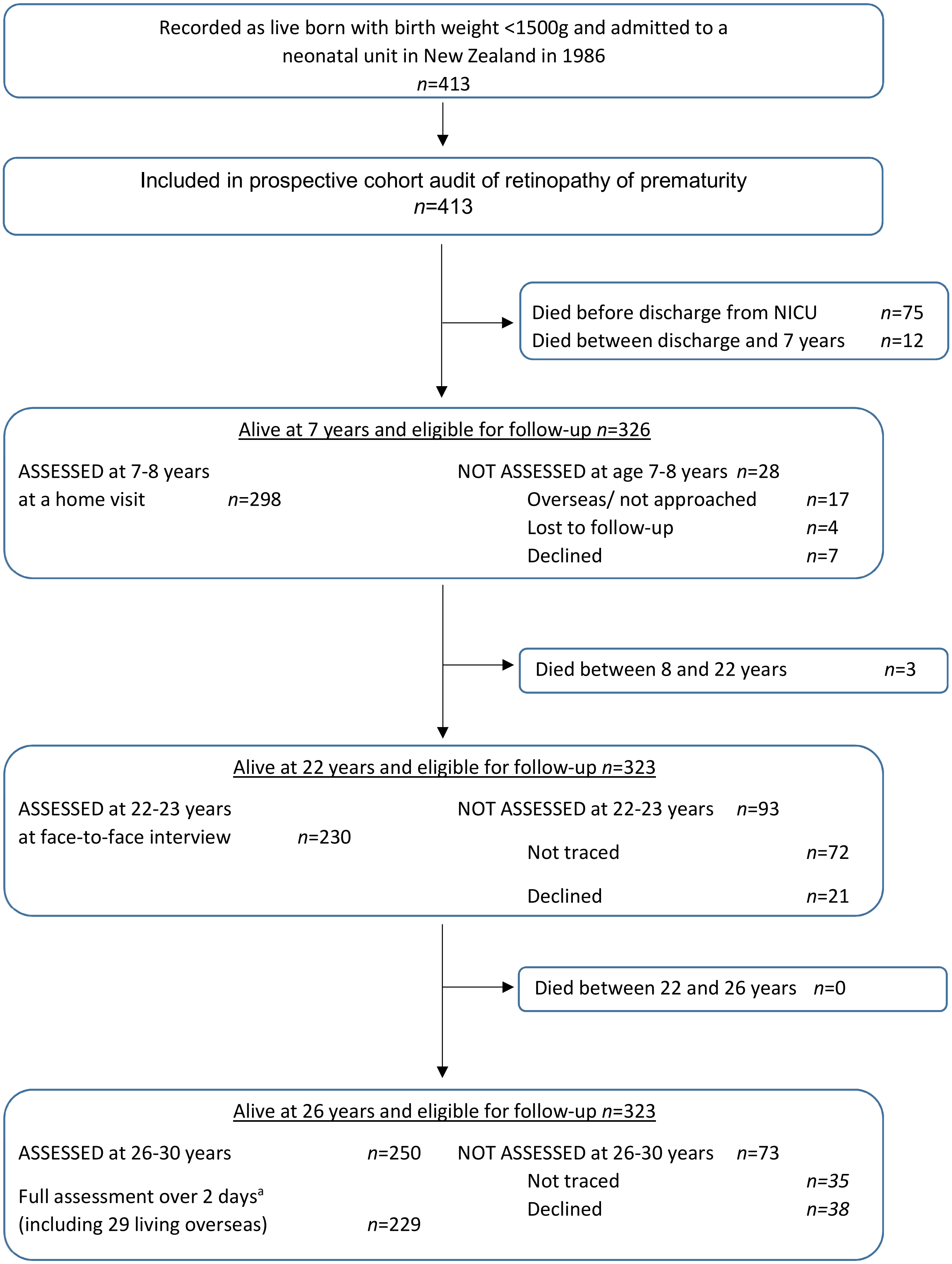

[a]Between February 2013 and November 2016. NICU: Neonatal intensive care unit.

Controls born at term in 1986: 69 recruited at 22-23 years, 39 seen again at 26-30 years plus 61 new recruits [Modified, with permission from Darlow BA, et al. Metabolic syndrome in very low birth weight young adults and controls: the New Zealand 1986 VLBW Study. *J Pediatr* 2019;206:128-33 (133.e1)]
