# Supplementary material for: Visuospatial outcomes of a prospective national cohort of young adults with very low birthweight
Source: Pediatr Res. 2025 Feb 7;98(5):1711–7. doi: 10.1038/s41390-025-03890-9 (PMC12602344; doi:10.1038/s41390-025-03890-9)
Supplement: Supplementary file 2 — Supplementary information [file 41390_2025_3890_MOESM2_ESM.pdf]

Visuospatial outcomes of a prospective national cohort of young adults with very low birthweight

Supplemental material

Details of cranial magnetic resonance imaging (MRI)

Supplemental Table S1. Comparison of the demographic and perinatal characteristics of the surviving VLBW cohort who were assessed on visuospatial outcomes with those not assessed.

Supplemental Table S2. Covariate adjusted effect sizes (Cohen's *d*, 95%*CI*s) for VLBW adults in comparison to controls by birth weight, gestation and ROP status of VLBW cohort.

Supplemental Table S3. Predictors of visuospatial composite score in the VLBW sample.

Supplemental Table S4. Comparison of demographic and perinatal characteristics of the surviving VLBW cohort who were assessed on MRI with those not assessed.

Supplemental Figure S1. New Zealand 1986 VLBW Adult Follow-up Study: Cohort flow chart

Details of cranial magnetic resonance imaging (MRI)

Imaging was conducted on a 3T General Electric HDxt scanner (GE Healthcare, Waukesha, WI) with an eight-channel head coil. The imaging protocol included: 1) a three-dimensional volumetric T1-weighted inversion-prepared spoiled gradient recalled echo sequence (SPGR, repetition time (TR)/echo time (TE)=6.6/2.8ms, inversion time (TI)=400ms, flip angle=15deg, acquisition matrix = 256 × 256 × 170, field of view (FOV) = 250 mm, slice thickness = 1 mm, voxel = 0.98 × 0.98 × 1.0 mm^3), 2) a T2 FLAIR (TE/TR = 116/9000 ms, TI = 2250 ms, flip angle = 90 deg, acquisition matrix = 320 × 320 × 32, reconstruction matrix = 512 × 512 × 32, FOV = 220 mm, axial slice thickness = 3 mm, gap = 1.5 mm, reconstructed voxel = 0.43 × 0.43 × 4.5 mm^3), and 3) a clinical T2-weighted image. Cortical thickness and surface area were estimated using FreeSurfer (v6.0, http://surfer.nmr.harvard.edu). Lobar estimates of cortical thickness were derived as a weighted average (by number of vertices) of individual cortical thickness estimates from regions defined by the Desikan-Killiany atlas.[1] Regions included in each lobe were as follows. *Frontal*: superior frontal, rostral and caudal middle frontal, pars opercularis, pars triangularis, pars orbitalis, lateral and medial orbitofrontal, precentral, paracentral, frontal pole, rostral and caudal anterior cingulate; *Parietal*: superior parietal, inferior parietal, supramarginal, postcentral, precuneus, posterior cingulate, isthmus; *Temporal*: superior, middle and inferior temporal, banks of the superior temporal sulcus, fusiform, transverse temporal, entorhinal, temporal pole, parahippocampal; *Occipital*: lateral occipital, lingual, cuneus, pericalcarine. Thickness estimates from right and left hemispheres were averaged to create a single average thickness per lobe; total surface area and volume per lobe were also calculated.

[1] Desikan RS, Ségonne F, Fischl B, et al. An automated labeling system for subdividing the human cerebral cortex on MRI scans into gyral based regions of interest. *Neuroimage.* 2006;31(3):968-980.

Supplemental Table S1. Comparison of the demographic and perinatal characteristics of the surviving VLBW cohort who were assessed on visuospatial outcomes with those not assessed

Measure	VLBW assessed (*N*=225)	VLBW not assessed[1] (*N*=98)	*P*[2]
Male, *%*	44.4	53.1	.15
Māori/Pacific Island ethnicity, *%*	31.1	33.7	.65
Birth weight (g), *mean (SD)*	1136 (235)	1182 (236)	.11
<1000g, *%*	27.1	23.5	.49
Gestation (wk), *mean (SD)*	29.3 (2.5)	29.2 (2.4)	.76
Extremely preterm (<28 wk), %	24.0	26.5	.63
Small for gestation[3], %	32.0	21.4	.054
Respiratory distress syndrome, %	54.2	61.2	.24
Bronchopulmonary dysplasia[4], %	19.6	24.5	.31
Antenatal corticosteroids, %	56.0	60.0	.48
Retinopathy of prematurity, %	18.7	21.4[5]	.56
Duration breast feeding (months)	4.5 (5.8)	5.0 (7.0)	.51
Any neurosensory disability (age 7-8 years)[6], %	21.9	33.7	.039
Mod/severe disability (age 7-8 years)[7], %	4.6	22.1	.001

[1] Includes 73 with no follow-up (35 not able to be contacted [13 known to be overseas], 38 contacted but declined); 21 not assessed in Christchurch but who consented to an interview; and 4 who were unable to complete the visuospatial assessment
[2] Comparisons of VLBW assessed and not assessed by *t*-test or chi square
[3] Birth weight <10th centile for gestation
[4] Oxygen requirement at 36 weeks post-menstrual age
[5] Includes 4 participants with bilateral stage 4 ROP and severe loss of vision who were assessed at 28 years but did not undergo test of visuospatial abilities
[6] Comparisons limited to those examined for disability at age 7-8: VLBW assessed (N=219) and not assessed (N=77)
[7] Moderate or severe disability at 7-8 years of age was defined as cerebral palsy in non-ambulant children or in ambulant children causing considerable limitation of movement, or bilateral sensorineural

deafness requiring hearing aids, or bilateral blindness, or an IQ score of >2 SD below the test mean (<70) on the Revised Wechsler Intelligence Scale for Children (WISC-R)

Supplemental Table S2. Covariate adjusted[1] effect sizes (Cohen's *d*, 95%*CI*s) for VLBW adults in comparison to controls by birth weight, gestation and ROP status of VLBW cohort

Measure	Birth Weight		Gestation		ROP Status		
	<1000g	1001-1499g	<28 Weeks	≥ 28 weeks	ROP Stage 2+	ROP Stage 1	No ROP
WASI II block design score (motor)	1.13 (.88, 1.38)	.62 (.37, .86)	1.09 (.83, 1.34)	.66 (.41, .90)	.94 (.54, 1.34)	.87 (.46, 1.29)	.73 (.51, .94)
Benton visuospatial processing score (non-motor)	.70 (.46, .95)	.51 (.27, .75)	.65 (.41, .89)	.54 (.30, .78)	.57 (.15, .99)	.67 (.17, 1.05)	.56 (.34, .78)
Brixton visuospatial sequencing score (non-motor)	.70 (.46, .95)	.29 (.05, .53)	.39 (.15, .63)	.41 (.17, .65)	.74 (.27, 1.21)	.20 (-.28, .69)	.39 (.15, .64)
Composite visuospatial processing score	1.07 (.82, 1.33)	.58 (.34, .83)	.91 (.66, 1.16)	.66 (.42, .91)	.95 (.52, 1.37)	.70 (.27, 1.14)	.70 (.48, .92)

[1] Adjusted for moderate or worse visual impairment, cerebral palsy, sex, ethnicity, maternal education, family socio-economic status
ROP: Retinopathy of prematurity; WASI II: Wechsler Adult Scale of Intelligence 2nd edition

Supplemental Table S3. Predictors of visuospatial composite score in the VLBW sample.

Predictor	Univariate Association (*r*)	Multiple Regression Model[1] (Standardised *β*)
Birth weight	.28***	.24***
Gestation	.13	
Small for gestation	-.05	
Male sex	.24***	.20**
Retinopathy of prematurity (stage)	-.07	
Antenatal corticosteroid use	.09	.11
Bronchopulmonary dysplasia	-.07	
Confirmed sepsis	-.02	
Intraventricular haemorrhage III/IV	-.04	
Severity of cerebral palsy	-.17*	-.13*
Maternal education	.14*	
Māori/Pacific Island ethnicity	-.21*	
Family socioeconomic status[2]	-.21*	-.22***
Moderate or worse visual impairment	.04	
Age at assessment	.10	
		R^2 = .17

* $p<.05$ ** $p<.01$ *** $p<.001$
[1] A p<.10 criterion was used for inclusion of predictors in the fitted regression model
[2] Socioeconomic status scored such that higher scores imply lower SES

Supplemental Table S4. Comparison of demographic and perinatal characteristics of the surviving VLBW cohort who were assessed on MRI with those not assessed

Measure	VLBW assessed on MRI (*N*=150)	VLBW not assessed on MRI (*N*=173)	*P*[1]
Age at scan (y), *mean (SD)*	28.5 (1.2)	-	-
Male, *%*	41.3	52.0	0.055
Māori/Pacific Island ethnicity, *%*	28.7	34.7	0.25
Birth weight (g), *mean (SD)*	1077 (238)	1213 (215)	<0.001
<1000g, *%*	38.0	15.6	<0.001
Gestation (wk), *mean (SD)*	28.8 (2.6)	29.6 (2.3)	0.002
Extremely preterm (<28 wk), *%*	35.3	15.6	<0.001
Apgar 5 min, *mean (SD)*	7.8 (1.9)	8.0 (1.7)	0.51
Small for gestation[2], *%*	30.7	27.2	0.49
Vaginal delivery, *%*	43.8	40.1	0.50
Confirmed sepsis, *%*	20.7	26.7	0.20
Antenatal corticosteroids *%*	54.7	59.5	0.38
Respiratory distress syndrome, *%*	56.7	56.1	0.91
Bronchopulmonary dysplasia[3], *%*	20.7	21.4	0.87
Retinopathy of prematurity, *%*	24.0	15.6	0.06
Any neurosensory disability (age 7-8 years), *%*	16.6	33.1	0.001
Mod/severe disability (age 7-8 years)[4], %	4.1	13.9	0.004

[1] Comparisons of VLBW assessed and not assessed by *t*-test or chi square

[2] Birth weight <10th centile for gestation

[3] Oxygen requirement at 36 weeks post-menstrual age

[4] Moderate or severe disability at 7-8 years of age was defined as cerebral palsy in non-ambulant children or in ambulant children causing considerable limitation of movement, or bilateral sensorineural

deafness requiring hearing aids, or bilateral blindness, or an IQ score of >2 SD below the test mean (<70) on the Revised Wechsler Intelligence Scale for Children (WISC-R).
